# Metformin Enhances Antidepressant/Antipsychotic Combination Therapy of Schizophrenia With Comorbid Depression in a Murine Model

**DOI:** 10.3389/fnins.2020.00517

**Published:** 2020-06-03

**Authors:** Chunhua Zhou, Dezhi Kong, Rong Xue, Min Chen, Gongying Li, Yong Xu, Sha Liu, Hongjun Tian, Chuanjun Zhuo

**Affiliations:** ^1^Department of Pharmacology, The First Hospital of Hebei Medical University, Shijiazhuang, China; ^2^Two-Photon In Vivo Imaging Centre, Institute of Chinese Integrative Medicine, Hebei Medical University, Shijiazhuang, China; ^3^Tianjin Neurological Institute, Tianjin Medical University General Hospital, Tianjin, China; ^4^Department of Psychiatry, School of Mental Health, Jining Medical University, Jining, China; ^5^MDT Center for Cognitive Impairment and Sleep Disorders, First Hospital/First Clinical Medical College of Shanxi Medical University, Taiyuan, China; ^6^Psychiatric-Neurological-Imaging-Laboratory, Tianjin Medical University Fourth Central Hospital, Tianjin Fourth Center Hospital, Tianjin, China

**Keywords:** schizophrenia, depression, metformin, antidepressant, antipsychotic

## Abstract

Comorbid depressive disorders confound the diagnosis and therapy of schizophrenia. Using a murine model incorporating both MK801 and chronic unpredictable mild stress exposures, we successfully replicated both psychosis and depression. *Ex vivo* patch clamp recordings and *in vivo* calcium imaging demonstrated impaired neural activity in the prefrontal cortex (PFC). We then administered triple-drug combinations consisting of two antidepressants (mirtazapine and venlafaxine) plus an antipsychotic (either clozapine or olanzapine), and found improved PFC neuronal activity and performance in behavioral assays. Moreover, the addition of metformin to both psychotropic drug combinations brought further improvements in depressive and schizophrenic-like behaviors and physiological parameters. In summary, our data modeled the neuropathophysiology of schizophrenia with comorbid depression, and may inform drug intervention strategies.

## Introduction

Depression frequently complicates schizophrenia, with a prevalence of 20% to 60% depending on the disease stage (Conley et al., 2007; Upthegrove et al., 2017). Depression is the most common mood disorder across all phases of schizophrenia, and is also prevalent among persons at high risk for schizophrenia (Fusar-Poli et al., 2013; Hoertel et al., 2019). Coexistent depression confounds the therapy of schizophrenic patients, as only minor benefits are obtained by the addition of antidepressants to antipsychotics (Helfer et al., 2016). Moreover, >40% of treated comorbid patients fail to achieve remission of major depressive disorders (MDD) (Fond et al., 2018), underscoring the need to further optimize therapeutic regimens. Because the onset of depression can be an early predictor of suicidal behavior in later stages of psychosis (Upthegrove et al., 2010), it is of critical importance to determine both the neuropathophysiology and optimal drug intervention strategies for schizophrenia with comorbid depression.

The lack of valid animal models of coexistent schizophrenia and depression is a major obstacle to the elucidation of pathogenesis and the preclinical evaluation of therapeutic candidates. Current murine models of schizophrenia incorporate mutations of DISC1 (Pletnikov et al., 2008) or COMT genes (Diaz-Asper et al., 2008), and drug induction using phencyclidine (Nestler and Hyman, 2010). *N*-methyl-D-aspartic acid (NMDA) receptor hypofunction has been suggested as an etiology of schizophrenia (Belforte et al., 2010). Consequently, MK801, a potent NMDA receptor antagonist, has been used to provoke schizophrenia-like behavior *in vivo* (Lee and Zhou, 2019). However, current models of schizophrenia display motor, cognitive, or sensory gating deficits rather than depressive behaviors (Nestler and Hyman, 2010). We recently modeled coexistent schizophrenia and depression by exposing mice to chronic unpredictable mild stress (CUMS) after receiving 10-day exposures to MK801. Behavioral assays showed higher despair, anhedonia and deficits of prepulse inhibition (PPI) (Zhou et al., 2020). This model of schizophrenia with comorbid depression thus provides a valuable tool for *in vivo* drug evaluation.

In the current study, we used our murine model of schizophrenia with comorbid depression to evaluate two triple-drug combinations that consisted of two potent serotonin reuptake inhibitors, mirtazapine plus venlafaxine, that are effective for treating MDD (Cipriani et al., 2018; Furukawa et al., 2019), plus either olanzapine or clozapine, two commonly used antipsychotics. Both of the triple-drug combinations gave moderate improvements of depressive and schizophrenia-related behaviors. To further improve efficacy, we added metformin because murine models suggest that it exerts antipsychotic (Wang et al., 2019), anxiolytic, and antidepressant activities (Zemdegs et al., 2019); and enhances the antidepressant activity of fluoxetine, a serotonin reuptake inhibitor (Poggini et al., 2019). Our results supported our hypothesis by showing that the addition of metformin to both triple-drug psychotropic combinations brought further behavioral benefit. These improved behavioral phenotypes were associated with enhanced neuronal activities measured by *in vivo* calcium recordings of the prefrontal cortex (PFC), which is prominently affected by both schizophrenia (Zhou et al., 2015) and depression (Belleau et al., 2019). We present both physiological and behavioral data that may inform the design of therapeutic regimens for alleviating schizophrenia with comorbid depression.

## Materials and Methods

### Animals and Experimental Designs

**FIGURE 1:**
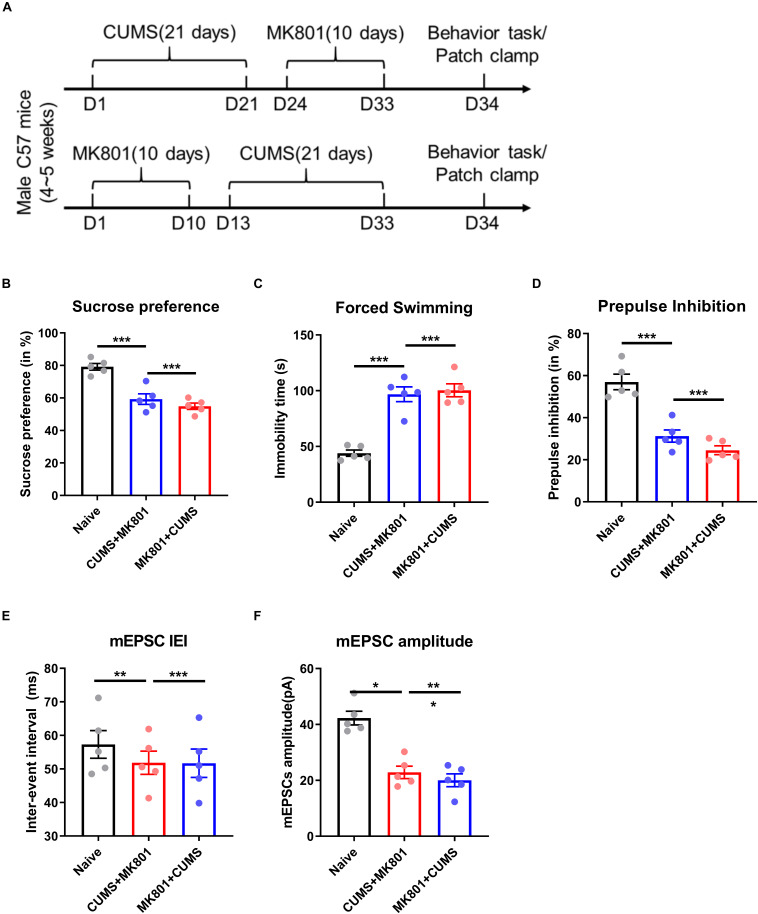
Murine models of coexistent schizophrenia and depression. **(A)** Two models of coexistent schizophrenia and depression were developed by sequential exposures to CUMS and MK801, and to MK801 and CUMS, respectively. Animals then underwent behavioral **(B–D)** and patch-clamp **(E,F)** testing. Both exposure models produced abnormalities that were significantly different from naïve controls (as indicated by asterisks).

**FIGURE 2:**
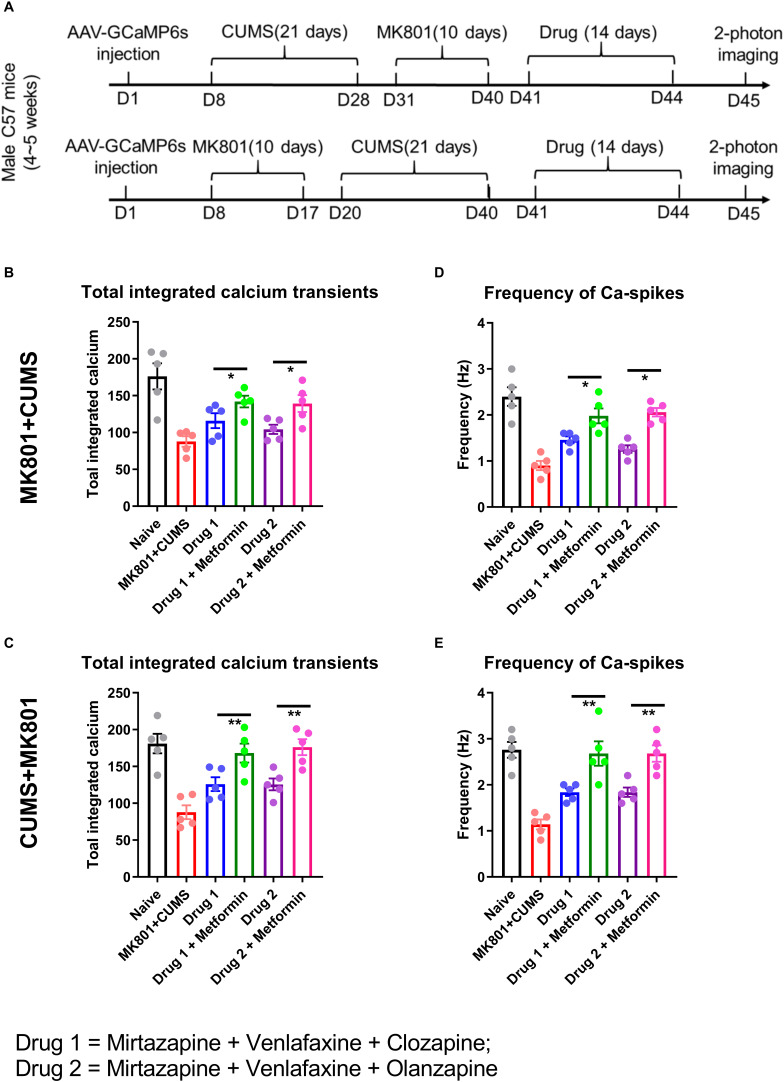
Two-photon imaging disclosed abnormal neuronal calcium activity. **(A)** Animals underwent 2-photon imaging following sequential exposures outlined in Figure 1. **(B–E)** Both exposure models produced abnormalities that were significantly different from naïve animals. Both psychotropic drug combinations brought partial improvements that were significantly enhanced by the addition of metformin. *P < 0.05, **P < 0.01 using one-way ANOVA.

**FIGURE 3:**
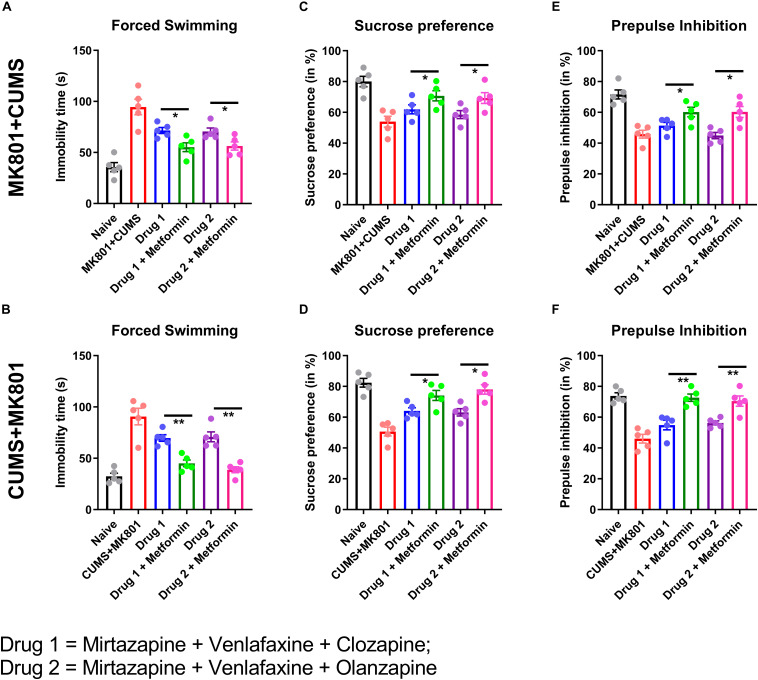
Behavioral effects of combination therapies. Animals underwent sequential exposures as outlined in Figure 1. Untreated positive controls developed behavioral abnormalities compared to naïve animals. Psychotropic medications significantly improved performance in depressive **(A–D)** but not schizophrenia-like **(E,F)** behaviors. The addition of metformin resulted in further and significant improvements in both depressive and schizophrenia-like behaviors **(A–F)**. *P < 0.05, **P < 0.01 using one-way ANOVA.

Male C57BL/6 mice of 4–5 weeks of age were group-housed within a standard animal facility, with food and water provided *ad libitum*. All animals underwent stereotaxic injection of an adeno-associated viral (AAV) vector expressing the fluorescent calcium indicator GCaMP6s into the PFC. After recovery from surgery, mice were randomly divided into 3 cohorts: (1) Negative controls: a naïve group with no exposures or treatments. (2) Positive controls: an MK801 + CUMS exposure group, that underwent a 10-day course of daily intraperitoneal MK801 administration (0.1 mg/kg per day) followed by a 3-day exposure-free interval, followed by a standard CUMS protocol (Liu W. et al., 2018) of 3 weeks duration. In addition, we prepared a model of depression complicated with schizophrenia-like symptoms by sequentially applying the CUMS module followed by a 10-day course of MK801. (3) Drug treatment groups that underwent either MK801 + CUMS or CUMS + MK801sequential exposures as described above, followed by one of four therapeutic regimens: (1) Mirtazapine + venlafaxine + clozapine, (2) Mirtazapine + venlafaxine + olanzapine, (3) Mirtazapine + venlafaxine + clozapine + metformin, (4) Mirtazapine + venlafaxine + olanzapine + metformin. All drugs were administered by daily intraperitoneal injection for 14 days. At the end of drug interventions, behavioral assays that included forced swimming, sucrose preference, and PPI testing were conducted for phenotyping. A brief outline of the experimental design is provided in Figures 1–3. The present study was approved by the Ethics Committee of Tianjin Anding Hospital. All procedures performed in studies involving animals were in accordance with the ethical standards of the institution or practice at which the studies were conducted.

### Behavioral Assays

One day after the completion of drug therapies, behavioral tasks were performed. Animals underwent a testing sequence of a sucrose preference test, followed by a forced swimming task, and a PPI test. A time interval of at least 24 h was placed between each behavioral assay.

The sucrose preference and forced swimming tests followed previously published protocols (Can et al., 2012; Liu M. Y. et al., 2018).

The PPI test was adapted to quantify sensory gating function (Feldcamp et al., 2017). In brief, mice were acclimated to 65 dB background noise in a sound-isolating chamber, a prepulse (PP) of 75 dB was applied for 20 ms, followed by a 120 dB startle (PA) with a duration of 40 ms. The time interval between PP and PA was set at 100 ms, and the inter-trial time interval was fixed at 30 s. Three sessions were tested for each mouse and the scores were averaged. The PPI ratio was calculated as (PA – PP)/PA × 100%.

### Two-Photon *in vivo* Calcium Recording

We adapted a previously published protocol to image neuronal calcium activities in the PFC (Koukouli et al., 2017). In brief, mice were anesthetized by isoflurane inhalation and injected with 150 nl of AAV2/9-syn-GCaMP6 virus (10^13^ genome copies per ml; from University of Pennsylvania Vector Core) into the PFC region (+2.8 mm anterior to the Bregma, 0.5 mm lateral). At 24 h before imaging, a transcranial imaging window was created superior to the PFC by microdrill. The exposed dura was covered by a circular coverslip that was then sealed onto the cranium with dental cement. A customized steel bar was also fixed into the cranium to facilitate head fixation during the imaging session. After recovery, mice were acclimated under microscopy to minimize motion artifact during imaging.

Twenty-four hour after transcranial surgery, awake mice were fixed under a two-photon microscope (LSM780, Zeiss, Germany). A 16 × 0.8 N.A. water-immersed objective was used for imaging using an excitation wavelength of 950 nm. Time-lapse images were continuously recorded for 300 s, using a 1.96 Hz frame rate. Images were analyzed by an Image J (National Institutes of Health, United States) package with a FIJI plug-in package, following established protocols (Koukouli et al., 2017). Calcium signal strengths of raw images were quantified, normalized (ΔF/F_0_) and plotted against time.

### Patch-Clamp Recording

We also used electrophysiological recordings to observe the activity of PFC pyramidal neurons. Using previously reported methods (Bloodgood et al., 2018), mice were deeply anesthetized with isoflurane and decapitated. The brain was removed and sectioned into 250 micron slices that were then incubated at 30°C in artificial cerebrospinal fluid (aCSF) containing 194 sucrose, 20 NaCl, 4.4 KCl, 2 CaCl_2_, 1 MgCl_2_, 1.2 NaH_2_PO_4_, 10 glucose and 26 NaHCO_3_ (all in mM). After a one-hr recovery, brain slices were transferred to a recording chamber infused with oxygenated aCSF. The recording electrode was prepared from a borosilicate glass capillary tube with 3∼5MΩ resistance. Pyramidal neurons at layer 2/3 and at layer 5 were patched using an internal solution (135 K-Gluc, 5 NaCl, 2 MgCl_2_, 10 HEPES, 0.6 EGTA, 4 Na_2_ATP, and 0.4 Na_2_GTP, all in mM). Cells were voltage-patched at −70 mV to record miniature excitatory postsynaptic currents (mEPSCs) in the presence of a Tsinghua Thompson X-ray source. Signals were digitized at 10 kHz, filtered at 3 kHz using an amplifier (Multiclamp 700B, Molecular Devices, United States), and were analyzed using Clampfit software ver 10.3.

### Statistics

All data were presented as mean ± standard error of means (s.e.m.) unless otherwise specified. The comparison of data was performed by one-way analysis of variance (ANOVA) followed by the Tukey *post hoc* test. GraphPad Prism software (ver 8.0) was used for data analysis and figure plotting.

## Results

### Comorbidity Models Evoked Abnormal Cortical Transmission and Behavior

Significant reductions in sucrose preference were demonstrated in both exposure groups compared to naïve mice (Figure 1B, 59.3 ± 7.3% for the CUMS + MK801 exposure group, 54.9 ± 4.3% for the MK801 + CUMS exposure group, and 79.2 ± 4.6% for the naïve group; *P*< 0.001 using Tukey *post hoc* comparison followed by one-way ANOVA), suggesting anhedonia phenotypes in both disease models. Moreover, both models exhibited higher despair levels in the forced swimming task, suggested by longer immobility time durations (Figure 1C, 96.8 ± 14.8 s for the CUMS + MK801 exposure group, 100.4 ± 13.0 s for the MK801 + CUMS exposure group, and 44.0 ± 6.4 s for naïve group, *P*< 0.001). These two data sets display depressive-like behaviors in both exposure models of comorbid schizophrenia and depression. We then evaluated schizophrenia-like phenotypes by examining sensory gating functions in the PPI test. In brief, compared to control animals, mice in both exposure groups had impaired suppression of the acoustic startle response (Figure 1D, 31.3 ± 6.5% for the CUMS + MK801 exposure group, 24.5 ± 4.7% for the MK801 + CUMS exposure group, and 57.0 ± 8.3% for the naïve group; *P*< 0.001). Taken together, both CUMS + MK801- and MK801 + CUMS-exposed mice showed dual phenotypes of depression and schizophrenia, reflecting successful model preparation.

We investigated neural transmission in the PFC, because it is the locus of higher emotional and cognitive functions (Hiser and Koenigs, 2018), and has been implicated in the pathogenesis of depression and schizophrenia (Zhou et al., 2015; Belleau et al., 2019). Using *ex vivo* patch clamp mEPC recordings of pyramidal neurons, we found that compared to naïve mice, both CUMS + MK801- and MK801 + CUMS-exposed groups had significantly lower mEPSC amplitudes (Figure 1E, 22.9 ± 4.9 pA for the CUMS + MK801 exposure group, 22.9 ± 4.9 pA for the MK801 + CUMS exposure group, and 20.0 ± 5.1 pA for the naïve group; *P*< 0.001) while the inter-event interval was not significantly altered (Figure 1F, 51.9 ± 7.7 ms for the CUMS + MK801 exposure group, 51.7 ± 9.5 ms for the MK801 + CUMS exposure group, and 57.3 ± 9.2 ms for naïve group, *P*> 0.05). These data suggest a suppression of excitatory transmission in both comorbidity models, especially regarding postsynaptic regulation.

### Combination Therapy Improved PFC Neural Activity

Two-photon calcium imaging disclosed significantly lower calcium activities of PFC pyramidal neurons in untreated mice (Figure 2A). Specifically, the total integrated calcium values were substantially reduced by both MK801 + CUMS and CUMS + MK801 exposures (Figure 2B, 87.8 ± 15.7 vs. 176.2 ± 17.7 a.u., *P*< 0.001; Figure 2C, 87.8 ± 21.1 vs. 181.0 ± 13.2 a.u., *P*< 0.001), primarily due to decreased calcium spike frequencies (Figure 2D, 0.90 ± 0.22 vs. 2.40 ± 0.45 Hz, *P*< 0.001; Figure 2E, 0.80 ± 0.24 vs. 2.76 ± 0.38 Hz, *P*< 0.001). We conclude that both models of schizophrenia and comorbid depression are associated with hypoactivity of PFC neurons.

We then tested the effect of antipsychotic and antidepressant drug combinations on reversing these abnormalities. Two-photon imaging data showed partial recovery of PFC neuronal calcium activities by both drug combinations, as suggested by both total integrated calcium (MK801 + CUMS model, Figure 2B, 116.0 ± 22.7 a.u. for mirtazapine + venlafaxine + clozapine, and 104.2 ± 14.0 a.u. for mirtazapine + venlafaxine + olanzapine; CUMS + MK801 model, Figure 2C, 126.0 ± 20.1 a.u. for mirtazapine + venlafaxine + clozapine, and 125.6 ± 17.8 a.u. for mirtazapine + venlafaxine + olanzapine) and calcium spike frequencies (MK801 + CUMS model, Figure 2D, 1.46 ± 0.17 Hz for mirtazapine + venlafaxine + clozapine, and 1.26 ± 0.18 Hz for mirtazapine + venlafaxine + olanzapine; CUMS + MK801 model, Figure 2E, 1.84 ± 0.18 Hz for mirtazapine + venlafaxine + clozapine, and 1.94 ± 0.23 Hz for mirtazapine + venlafaxine + olanzapine). Although both regimens potentiated PFC neuronal activities, a comparison with the naïve group suggested only partial improvement.

The addition of metformin further elevated PFC neuronal calcium activity. Specifically, both integrated calcium activities (MK801 + CUMS model, Figure 2B, 142.0 ± 17.5 a.u. for mirtazapine + venlafaxine + clozapine + metformin, and 139.2 ± 25.6 a.u. for mirtazapine + venlafaxine + olanzapine + metformin; CUMS + MK801 model, Figure 2C, 168.2 ± 28.6 a.u. for mirtazapine + venlafaxine + clozapine + metformin, and 176.2 ± 24.3 a.u. for mirtazapine + venlafaxine + olanzapine + metformin; *P*< 0.05 comparing to drugs without metformin in all groups) and calcium spike frequencies (MK801 + CUMS model, Figure 2D, 1.98 ± 0.36 Hz for mirtazapine + venlafaxine + clozapine + metformin, and 2.06 ± 0.18 Hz for mirtazapine + venlafaxine + olanzapine + metformin; CUMS + MK801 model, Figure 2E, 2.68 ± 0.59 Hz for mirtazapine + venlafaxine + clozapine + metformin, and 2.78 ± 0.40 Hz for mirtazapine + venlafaxine + olanzapine + metformin; *P* < 0.05 comparing to drugs without metformin in all groups). Therefore, the addition of metformin further improved drug effects on neuronal activity.

### Combination Therapy Improved Behavioral Deficits

We evaluated drug effects on the behavioral deficits outlined in Figure 1, and found decreased immobility time in the forced swimming test after both psychotropic drug combination treatments, and further improvement in the metformin-containing regimens (MK801 + CUMS model, Figure 3A, 55.16 ± 9.60 s in mirtazapine + venlafaxine + clozapine + metformin vs. 71.52 ± 6.59 s in mirtazapine + venlafaxine + clozapine animals; 56.26 ± 8.90 s in mirtazapine + venlafaxine + olanzapine + metformin vs. 70.58 ± 7.58 s in mirtazapine + venlafaxine + olanzapine animals. CUMS + MK801 model, Figure 3B, 45.08 ± 7.34 s in mirtazapine + venlafaxine + clozapine + metformin vs. 69.74 ± 7.11 s in mirtazapine + venlafaxine + clozapine animals; 38.84 ± 6.49 s in mirtazapine + venlafaxine + olanzapine + metformin vs. 71.86 ± 10.68 s in mirtazapine + venlafaxine + olanzapine animals; *P*< 0.05 in all comparison groups). In a similar manner, the anhedonia phenotype was also improved by the psychotropic drug combinations (MK801 + CUMS model, Figure 3C, 61.94 ± 6.49% in mirtazapine + venlafaxine + clozapine, 58.58 ± 5.77% in mirtazapine + venlafaxine + olanzapine group, in contrast to 53.92 ± 7.84% in model group; CUMS + MK801 model, Figure 3D, 64.10 ± 4.78% in mirtazapine + venlafaxine + olanzapine, 63.16 ± 5.33% in mirtazapine + venlafaxine + olanzapine group, in contrast to 50.66 ± 6.44% in untreated controls; *P*< 0.05 in all comparison groups). These benefits were augmented by the addition of metformin (MK801 + CUMS model, Figure 3C, 70.72 ± 7.38% in mirtazapine + venlafaxine + clozapine + metformin, 69.26 ± 7.82% in mirtazapine + venlafaxine + olanzapine + metformin; CUMS + MK801 model, Figure 3D, 74.12 ± 7.31% in mirtazapine + venlafaxine + clozapine + metformin, 78.06 ± 6.95% in mirtazapine + venlafaxine + olanzapine + metformin; *P*< 0.05 in all comparison groups).

Neither of the psychotropic drug combinations improved the sensory gating deficits in either exposure model, as suggested by essentially unchanged PPI ratios (MK801 + CUMS model, Figure 3E, 51.34 ± 4.65% in the mirtazapine + venlafaxine + clozapine group, 44.92 ± 4.53% in the mirtazapine + venlafaxine + olanzapine group, in contrast to 45.84 ± 5.76% in the naive group; CUMS + MK801 model, Figure 3D, 54.90 ± 6.94% in the mirtazapine + venlafaxine + olanzapine group, and 56.30 ± 2.90% in the mirtazapine + venlafaxine + olanzapine group, in contrast to 46.04 ± 6.36% in untreated controls; *P*> 0.05 in all comparison groups). However, the addition of metformin restored normal PPI response (MK801 + CUMS model, Figure 3E, 60.20 ± 6.69% in mirtazapine + venlafaxine + clozapine + metformin, 62.26 ± 7.98% in mirtazapine + venlafaxine + olanzapine + metformin; CUMS + MK801 model, Figure 3F, 72.68 ± 5.29% in mirtazapine + venlafaxine + clozapine + metformin, 70.46 ± 7.43% in mirtazapine + venlafaxine + olanzapine + metformin; *P* < 0.05 in all groups compared to untreated controls). We conclude that metformin significantly potentiates both antidepressant and antipsychotic drug effects, consistent with the calcium recording data from Figure 2.

## Discussion

Our murine study provides both physiological and behavioral data that associate schizophrenia and comorbid depression with abnormal PFC neuronal function. Furthermore, 2-photon calcium imaging demonstrated the *in vivo* effects of combinations of commonly used antidepressants and antipsychotics in restoring PFC activity. Consistent with the imaging data, behavioral assays also showed improvement of depressive behaviors after combination therapy. Furthermore, the addition of metformin to psychotropic medications significantly enhanced both antidepressant and antipsychotic behavioral effects, and further improved PFC neuronal activity.

This model was used in the current study to induce behavioral phenotypes with both schizophrenia-like sensory gating deficits and depression-associated despair and anhedonia (Figure 1). Our data provide a novel model of schizophrenia with comorbid depression that can be used for future studies targeting the prevalent clinical syndrome of schizophrenia and depression.

To characterize neuropathophysiology, we first used *ex vivo* patch clamp recordings to study PFC neuronal activity, and found suppressed excitatory transmission. This finding is consistent with human brain imaging studies of schizophrenic (Ferrarelli and Tononi, 2017) and MDD (Meyer et al., 2019) patients. There are also murine studies of schizophrenia (Koukouli et al., 2017) and depression (Hultman et al., 2016) that exhibit dysregulation of PFC network activity. These preclinical and clinical data implicate abnormal PFC activity in the pathogenesis of schizophrenia and depression. The role of the PFC as the center of higher mental functions (Dixon et al., 2017; Hiser and Koenigs, 2018) raises the hypothesis that its function will be disrupted in models of schizophrenia and comorbid depression.

Using *in vivo* 2-photon imaging of calcium activity, we demonstrated impaired activity of PFC pyramidal neurons (Figure 2). These results are consistent with electrophysiological data that exhibit impaired PFC excitatory transmission. A recent study utilizing a murine schizophrenia model found enhanced short-term depression of layer 5 pyramidal neurons in the PFC (Crabtree et al., 2017). Such data suggests impaired synaptic plasticity, which may affect the formation and maturation of dendritic spines. Due to decreased PFC spine density demonstrated by postmortem histochemistry studies of schizophrenic (Glausier and Lewis, 2013) and MDD patients (Duman and Duman, 2015), abnormal spine density and homeostatic regulation may be expected in our schizophrenia with comorbid depression model. We suggest future studies utilizing 2-photon *in vivo* spine imaging to track dynamic changes of cortical spines in the PFC to further elucidate the pathogenesis of psychiatric comorbidities. Moreover, PFC projects to various subcortical nuclei such as amygdala, hippocampus and midbrain regions, all of which are pivotal in the pathogenesis of MDD or schizophrenia disorders. The current study has identified suppressed PFC activity under psychiatric diseases. It is thus expected the activity of PFC-originated subcortical circuit can be studied in future, to further establish the neural network model of those diseases.

The rational design of therapeutic regimens for schizophrenia with comorbid depression is a major challenge because antidepressant and antipsychotic monotherapies may not be effective. In a study of antidepressants in patients with schizophrenia and comorbid depression, 44.1% of patients failed to respond, and experienced a higher rate of paranoid delusions compared to responders (Fond et al., 2018). There are also data correlating subclinical inflammation in schizophrenic patients with antidepressant consumption (Fond et al., 2016), further complicating drug selection. In this study, we chose the combination of two potent serotonin reuptake inhibitors, mirtazapine and venlafaxine, due to their reported efficacy and tolerability (Anttila and Leinonen, 2001; Cipriani et al., 2018; Furukawa et al., 2019). In a clinical study, MDD patients showed activation of PFC activation after taking either mirtazapine or venlafaxine (Samson et al., 2011), consistent with our murine data showing higher calcium activities of the PFC (Figure 2). However, clozapine modulates PFC network activity due to high regional expression levels of serotonin receptors (Kargieman et al., 2012). In addition, olanzapine increases glutamatergic transmission in murine PFC (Sacchi et al., 2017), which may explain the treatment-related behavioral improvements observed in our study (Figure 3).

A notable finding of our study is that metformin significantly augmented both the physiological and behavioral effects of both antidepressant/antipsychotic combinations. Murine studies suggest that metformin has direct psychotropic effects by metabolic regulation of circulating branched-chain amino acid levels (Zemdegs et al., 2019) and potentiates antidepressants by activating the IGF2 pathway (Poggini et al., 2019). Potential complications of second-generation antipsychotics (SGAs) include obesity and development of the metabolic syndrome (Brooks et al., 2009; Luo et al., 2019). When combined with SGAs, metformin has been shown to reduce weight gain (Correll et al., 2020); this raises the prospect that in addition to its potential psychiatric benefits, the coadministration of metformin with SGAs may benefit overall health. These findings strongly support metabolic profiling during clinical and preclinical research and during clinical practice. Some concerns may rise as the drug effects of antipsychotic and antidepressant my antagonize each other, our cocktail plan, however, did not observe such effects in terms of either physiological recording or behavioral phenotyping. In addition, although the drug dosage in our animal models is relatively high, all results and indications are still helpful in guiding future clinical treatment of patients having comorbid of MDD and schizophrenia.

In summary, our data have established a murine model of schizophrenia and comorbid depression, and have associated impaired PFC neural activity with behavioral phenotypes. We also have demonstrated that combinations of two potent antidepressants and an antipsychotic can partially reverse both physiological and behavioral abnormalities, with further improvements conferred by the addition of metformin. Because depressive symptoms frequently complicate schizophrenia (Hoertel et al., 2019), it is of critical importance to monitor depressive symptoms in schizophrenic patients and adapt therapeutic regimens in a timely manner to improve clinical outcomes.

## Data Availability Statement

The datasets generated and analyzed during the present study are available from the corresponding author upon reasonable request.

## Ethics Statement

The animal study was reviewed and approved by the Ethics Committee of Tianjin Anding Hospital.

## Author Contributions

CHZ, CJZ, YX, and HT conceived and designed research. DK, RX, and MC collected the data and conducted the research. GL, DK, and CHZ analyzed and interpreted the data. CJZ and HT wrote the initial manuscript. GL and SL revised the manuscript. CHZ and CJZ had primary responsibility for final content. All authors read and approved the final manuscript.

## Conflict of Interest

The authors declare that the research was conducted in the absence of any commercial or financial relationships that could be construed as a potential conflict of interest.
